# Endometrioid carcinomas with sex cord-like formations and hyalinization: spontaneous pregnancy after conservative treatment

**DOI:** 10.1186/s12884-023-06219-8

**Published:** 2024-01-04

**Authors:** Qujia Gama, Shuhan Luo, Pengfei Wu, Lulu Wang, Sijia Liu, Hongwei Zhang, Li Sun, Yiqin Wang, Min Yu, Xiaojun Chen, Weiwei Shan, Xuezhen Luo

**Affiliations:** 1 Department of Gynecology, Obstetrics and Gynecology Hospital of Fudan University, 128 Shenyang Road, Yangpu District, Shanghai, 200090 P.R. China; 2 Shanghai Key Laboratory of Female Reproductive Endocrine-Related Diseases, Shanghai, 200090 China; 3 Department of Cervix, Obstetrics and Gynecology Hospital of Fudan University, Shanghai, 200090 China; 4 Department of Ultrasound, Obstetrics and Gynecology Hospital of Fudan University, Shanghai, 200090 China; 5 Department of Pathology, Obstetrics and Gynecology Hospital of Fudan University, Shanghai, 200090 China; 6 Department of Assisted Reproduction, Obstetrics and Gynecology Hospital of Fudan University, Shanghai, 200090 China

**Keywords:** Fertility-sparing treatment, Corded and hyalinized endometrioid adenocarcinoma (CHEC)

## Abstract

Endometrioid carcinoma with sex cord-like formations and hyalinization of the uterine corpus, or corded and hyalinized endometrioid adenocarcinoma (CHEC), is a rare morphological variant of endometrioid carcinoma, for which there is limited literature and few cases reports. Most researchers tend to consider CHEC as a low-grade cancer with a favorable prognosis. Full-staging surgery is the primary choice for this disease, and no case of CHEC has been previously reported to be treated conservatively. Here, we present the following case to explore the possibility of fertility-preserving treatment for young women with CHEC. A 23-year-old nulliparous patient diagnosed with presumed stage IA CHEC received fertility-sparing treatment at the Obstetrics and Gynecology Hospital of Fudan University and got a complete response (CR) after 10 months of conservative treatment. The patient subsequently became pregnant spontaneously, successfully conceived, and gave birth to a healthy male neonate without any sign of recurrence during 37 months follow-up after CR. The patient’s postpartum follow-up is continuing. Presently, CHEC is not included in the fertility-sparing field of any available guidelines. This case indicates that fertility-sparing treatment may be an option for highly selected patients with CHEC. Continuous follow-up remains mandatory to observe long-term outcomes.

## Introduction

Corded and hyalinized and spindled endometrioid carcinomas of the uterine corpus (CHEC) are rare variants of endometrial endometrioid adenocarcinoma (EEC) that were first described in 2005 by Murray et al. [1] and are characterized by cords of low-grade epithelial cells within a hyalinized stroma or spindled epithelial cells in addition to the classical endometrioid adenocarcinoma components. CHEC has been considered a low-grade EEC, with most cases being classified as grade 1 or 2 [1–5]. The standard treatment is staging surgery, but for young nulliparous females, surgery constitutes a complete loss of fertility and may be unacceptable for them. Previous studies [6–8] have shown that highly selected patients with low-grade presumed stage IA EC can undertake fertility-sparing treatment with good outcomes, which has been approved by the guidelines [9, 10].

According to the available English literature, a total of 56 patients with CHEC have been reported [1–5]. Patients with CHEC are generally diagnosed at a younger age (mean, 48.8 years) than patients with typical EEC (mean, 63 years [11]), and patients with CHEC generally harbor early clinical stage with good prognosis, which is similar to low-risk EEC. Theoretically, fertility-sparing treatment may be worth trying in young patients with CHEC who desire to preserve their fertility [1, 2].

Due to their “biphasic” morphologies, CHECs are often confused with other neoplasms, such as malignant mullerian mixed tumor (MMMT), sertoliform EECs, low-grade endometrial stromal sarcomas (LGESS) with sex cord-like patterns, and uterine tumors resembling ovarian sex cord tumors (UTROSCTs). So it is of vital importance to make a precise pathological diagnosis before moving forward with proper treatment and to avoid overtreatment, especially in young females of childbearing age.

Here we report the first case of CHEC, in which the patient successfully conceived and delivered following conservative treatment. Our case provides experience in treating patients with CHEC conservatively; however, future studies are necessary to further understand this disease.

## Case description

**Fig. 1:**
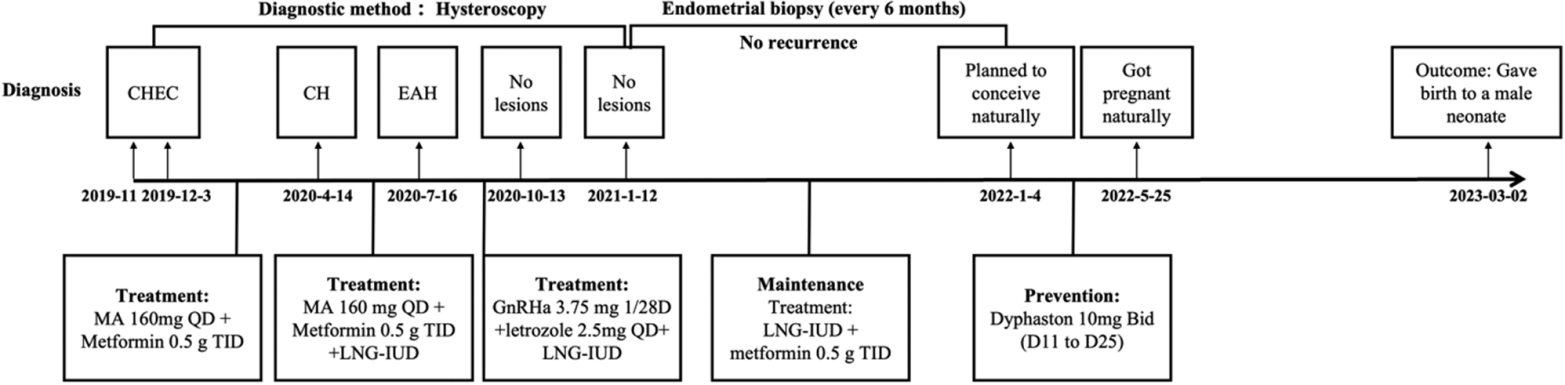
Timeline overview. CHEC, corded and hyalinized endometrioid adenocarcinoma; CH, complex hyperplasia; EAH, endometrial atypical hyperplasia; MA, megestrol acetate; GnRHa, gonadotropin-releasing hormone agonist; LNG-IUD, Levonorgestrel-releasing intrauterine device

**Fig. 2:**
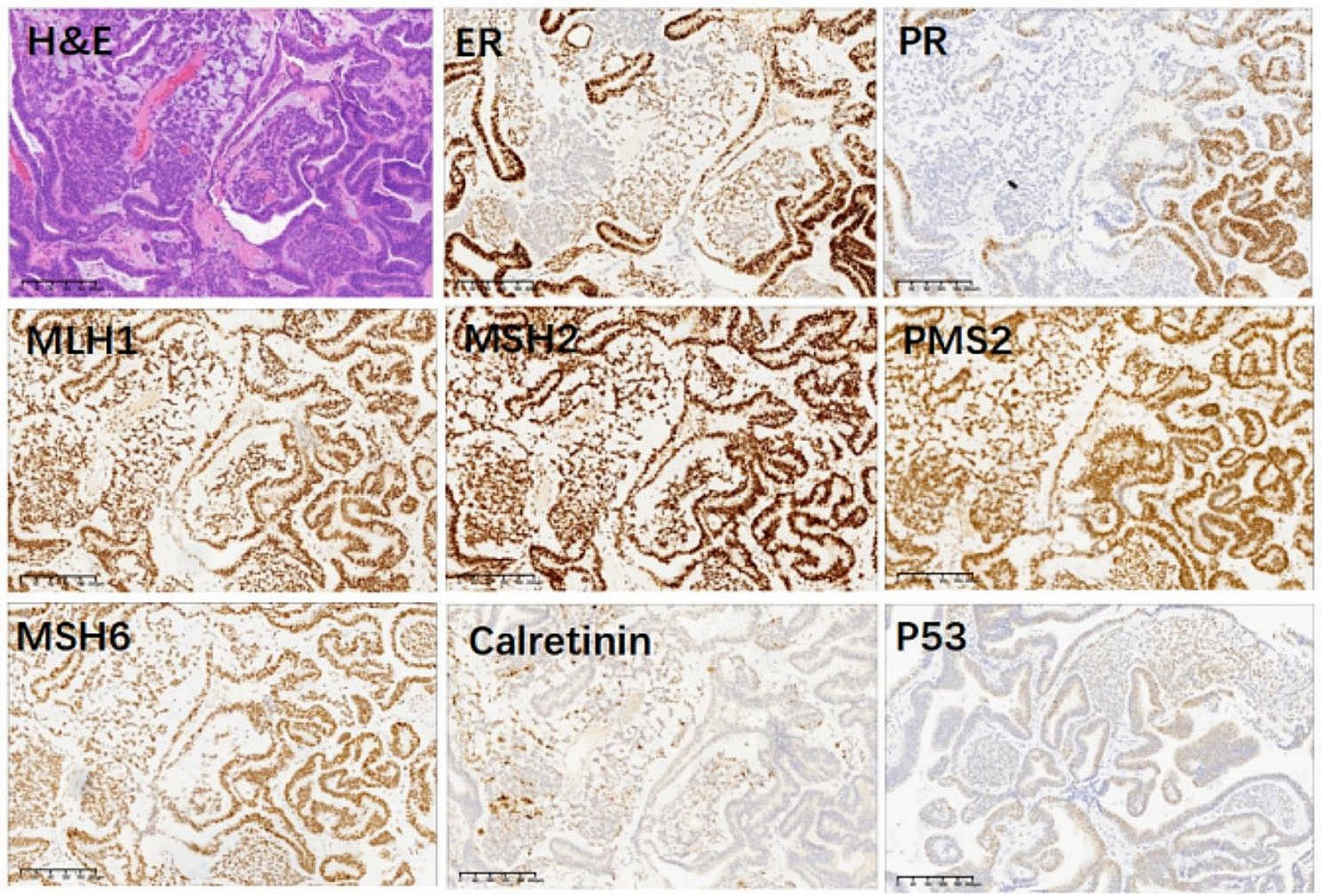
Immunohistochemistry staining of primary corded and hyalinized grade 1 endometrioid adenocarcinoma (CHEC) pathology before initiating conservative treatment. The glandular epithelium demonstrated strong staining of ER and PR and negative expression of Calretinin, while the squamous and corded components displayed negative expression of ER and PR and positive expression of Calretinin. All areas showed positive expression of Lynch screening indicators (MLH1, MSH2, PMS2 and MSH6) and wild type of P53. H&E, Hematoxylin and Eosin; ER, estrogen receptor; PR, progesterone receptor

The 27-year-old nulliparous female suffered from CHEC, conceived naturally, and successfully bore a male neonate after fertility-sparing treatment. The treatment timeline is shown in Fig. 1. The patient came to the outpatient clinic of the Obstetrics and Gynecology Hospital of Fudan University in October 2019 with the complaint of having had menometrorrhagia for months; an ultrasound examination indicated a hyperechoic area (46*18*11 mm) in the uterine cavity extending into the cervical canal. The patient had received right oophorectomy as a child in a local hospital due to a benign disease and had no family history of tumors. The patient underwent a hysteroscopic examination and was diagnosed with endometrioid carcinoma grade 1 with squamous, local cord-like formations and hyalinization with positive expressions of estrogen receptor (ER) and progesterone receptor (PR) and negative expression of Calretinin in the area of endometrioid adenocarcinoma, as well as negative expressions of ER and PR and positive expression of Calretinin in the area of sex cord-like formations (Fig. 2). The patient refused a hysterectomy and demanded fertility-sparing treatment and was fullyinformed that there was no available data on conservative treatment for CHEC. The patient and the patient’s family were counseled extensively on the advantages, disadvantages, and risks of surgery and conservative treatment. They provided written informed consent; then, primary assessment was performed before initiating fertility-sparing treatment.

The patient’s basic information; metabolic conditions; serum tumor biomarkers; pelvic examination; imaging assessment, including ultrasound scanning and pelvic magnetic resonance imaging (MRI) and abdominal computed tomography (CT) scanning; and hysteroscopic examination were fully assessed. The body mass index (BMI) of the patient was 20.70 kg/m^2^, and the blood pressure (BP) was normal. Fasting blood examination indicated no abnormal findings in liver and renal functions, fasting lipids, serum tumor biomarkers (CA125), blood glucose, or fasting insulin (FINS). Homeostasis model assessment-insulin resistance (HOMA-IR) was calculated as FBG (mmol/L) × FINS (µU/mL)/22.5. The patient had no insulin resistance, with a HOMA-IR value of 1.68, and no signs of metabolic syndrome (MS). The patient’s AMH was 0.8 ng/ml, indicating diminished ovarian reserve (DOR); however, the patient’s other hormonal levels (including estradiol, progesterone, testosterone, follicle-stimulating hormone (FSH), and luteinizing hormone (LH)) were in normal. Abdominal enhanced CT indicated a small angioma in the liver and follow-up was advised. The pelvic enhanced MRI indicated residual lesions in the uterine cavity and cervical canal, as well as a left ovarian simple cyst. No extrauterine lesions, myometrial invasion, nor metastasis were found.

**Fig. 3:**
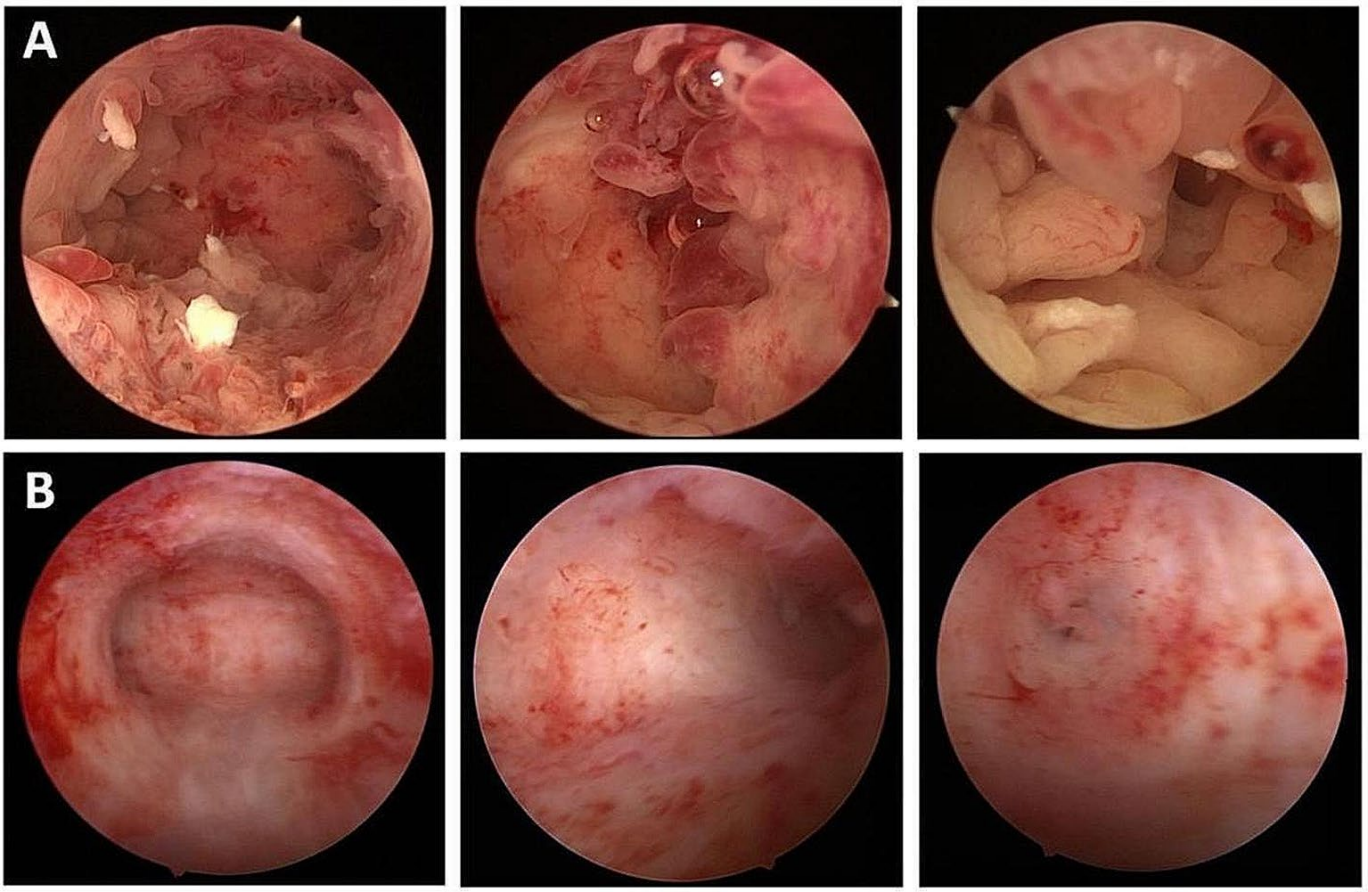
The patient’s uterine cavity images captured during the hysteroscopy. Each group of photos displays the entire uterus cavity, the left uterine horn, and the right uterine horn from left to right. (**A**) The patient had the first hysteroscopy performed in our clinic on December 3, 2019, which showed that diffused cancerous lesions lay in the uterine cavity as well as the bilateral horns. (**B**) The hysteroscopy view captured on October 13, 2020. No visible lesions were found which was also confirmed by postoperative pathology and the patient achieved primary complete response

**Fig. 4:**
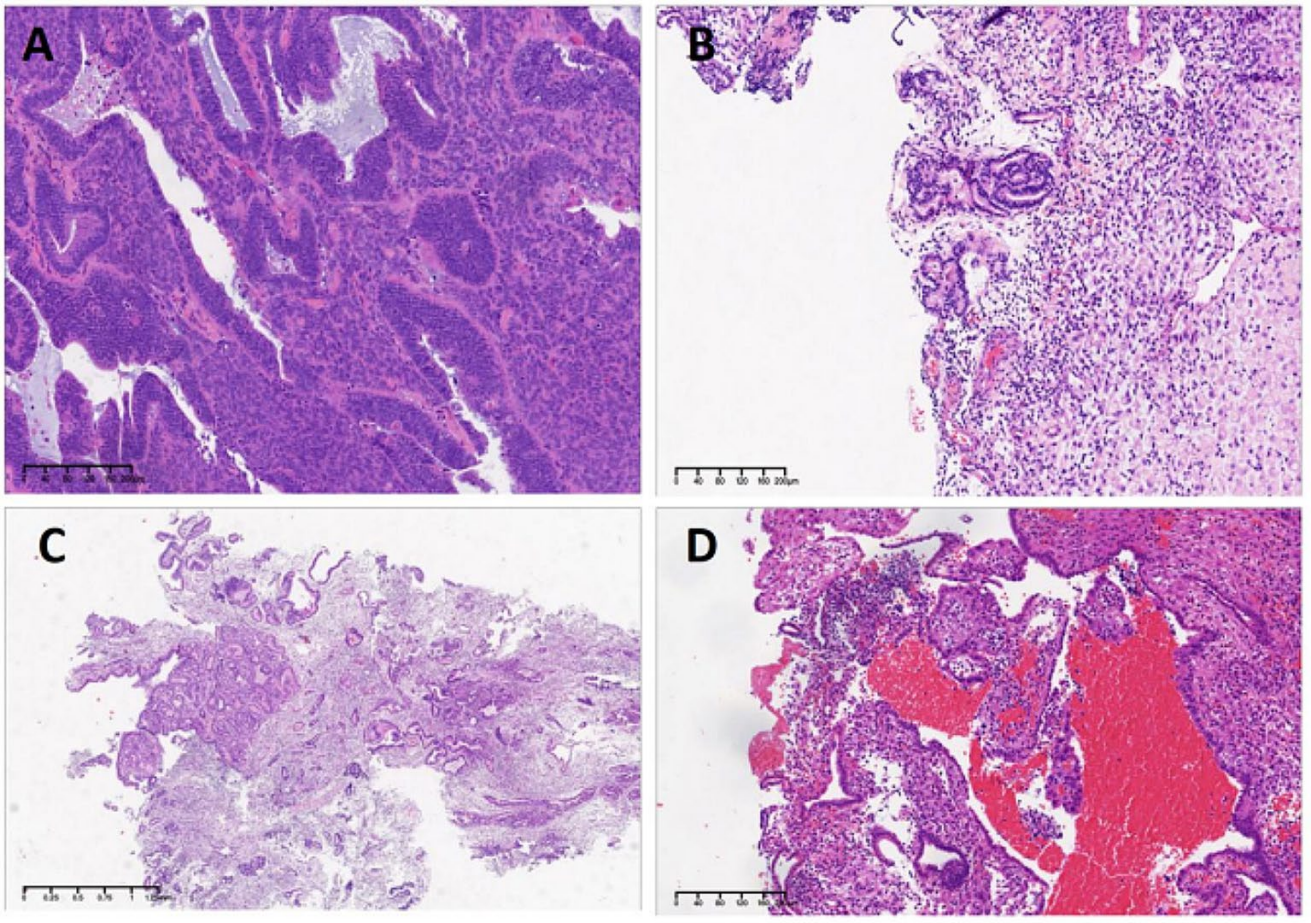
Endometrial pathology evaluated by hysteroscopy during conservative treatment. (**A**) Pathology of hysteroscopy on December 3, 2019, showed corded and hyalinized endometrioid adenocarcinoma (CHEC). (**B**) Pathology of hysteroscopy on April 14, 2020, indicated focal complex hyperplasia (CH). (**C**) Pathology of hysteroscopy on July 23, 2020, indicated endometrial atypical hyperplasia (EAH). (**D**) Pathology of hysteroscopy on October 2020, indicated no lesions. H&E, Hematoxylin and Eosin

**Fig. 5:**
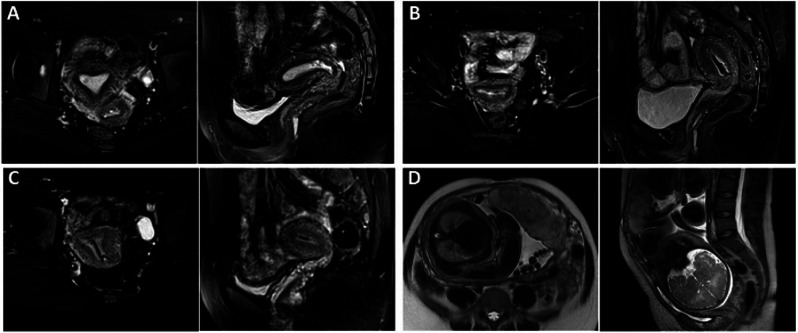
Pelvic MRI scans obtained from the patient. From left to right, each group of images shows the uterus on axial and sagittal T2-weighted imaged by MRI. (**A**) Enhanced MRI on November 29th, 2019 indicated residual lesions after the first hysteroscopy. (**B**) Enhanced MRI on July 14th, 2020, indicated thin endometrium; no significant lesions were found. (**C**) Enhanced MRI on August 14th, 2021 and (**D**) non-enhanced MRI on February 7th, 2023 before delivery showed no significant lesions

Then, a comprehensive hysteroscopic assessment was performed and residual lesions were removed as much as possible (Fig. 3A). The pathology was consistent with the preoperative diagnosis (Fig. 4A). Lynch syndrome screening (Fig. 2) and germline genetic screening were performed and no positive indications were reported. There was no available reference on conservative treatment on CHEC, therefore, we tried and adopted high-efficacy, high-dosage, progesterone-based treatment for the patient according to conservative treatment of presumed stage IA EEC. Oral megestrol acetate 160 mg daily and metformin 0.5 g three times per day were also prescribed for the patient after the hysteroscopic assessment in December 2019. After multidisciplinary discussion, Levonorgestrel-releasing intrauterine device (LNG-IUD) was inserted into the uterine cavity during the patient’s second hysteroscopy on April 14, 2020, after initiating conservative treatment considering the specificity of pathological types. Endometrial pathology was assessed every 3–4 months by hysteroscopy. Pelvic magnetic examinations were performed during treatment (Fig. 5).

After 4 months of therapy, a hysteroscopic assessment was performed and postoperative pathology indicated focal complex hyperplasia (CH) (Fig. 4B). After 7 months of therapy, the hysteroscopic pathology was endometrial atypical hyperplasia (EAH) (Fig. 4C). Given that the patient may not have been sensitive to progesterone therapy, the operation was suggested again, and the patient refused again. The patient’s medication regimen was subsequently changed to intramuscular injection of gonadotropin-releasing hormone agonist (GnRHa) 3.75 mg 1/28D plus oral letrozole 2.5 mg daily, and the LNG-IUD was kept. No endometrial lesions were indicated after 3 months of the new regimen therapy, and the patient had the first complete response (CR) (Figs. 3B and 4D). Then, the patient had a second CR on October 13, 2020. LNG-IUD plus metformin 0.5 g three times per day was suggested as the maintenance therapy. Endometrial biopsies were performed every 6 months after CR, and no signs of recurrence were found.

On January 2022, approximately 15 months after the second CR, the patient received family planning services refused assisted reproductive technology, and wanted to try natural pregnancy. Accordingly, the patient had the LNG-IUD removed and began taking duphaston 10 mg twice daily from day 11 to day 25 of the cycle for endometrial protection. The patient’s hysterosalpingography examination indicated intrauterine adhesion, obstruction of the isthmus of the right fallopian tube, and partial obstruction of the left tube. Considering the patient’s limited ovarian reserve and poor oviduct conditions, in vitro fertilization and embryo transfer (IVF-ET) were again intensely recommended. However, the patient refused and tried to conceive naturally. Fortunately, the patient succeeded and became pregnant after 3 months. At 40 weeks one day gestation, the patient successfully gave birth to a male neonate (3500 g) naturally with episiotomy on March 2, 2023. The patient underwent artificial placental dissection due to placenta adhesion. Hysteroscopy examination was performed because of retained placental tissue at 3 months postpartum and no lesions were found. The patient ended a seven-month lactation period in October. LNG-IUD insertion was completed in November as maintenance therapy.No recurrence was observed. The long-term follow-up is continuing.

## Discussion

Here, we report the first CHEC case, in which the patient successfully conceived and delivered after receiving conservative treatment. Our report mainly highlights the feasibility of reproductive organ preservation for patients with CHEC. Uterine preservation may be feasible even in patients with CHEC under intensive monitoring.

The CHEC is characterized by cords of epithelial cells with or without spindle cells embedded in a prominent hyalinized to myxoid stroma and exhibits “biphasic” morphology [1]. According to previous studies [1–5], most histological grades of the endometrioid adenocarcinoma components were grades 1–2 and associated with squamous epithelium. Sex cord structure accounted for 10–60%, which sometimes shows an epithelial-like morphology, similar to adenocarcinoma cells, while at other times demonstrating fusiform, similar to mesenchymal components. The chromatin of sex cord cells is relatively delicate, and the karyotypes can be classified into mild, moderate atypia, and severe atypia, with severe atypia being rare. Sex cord structures are often accompanied by hyalinization, and sex cord cells may appear as clusters or single cells embedded in hyalinization structures; moreover, some hyalurous mesenchyme may form small nodules (mimicking cartilage formation) or frequently show osseous metaplasia. Due to the “biphasic” morphology of CHEC, precise pathological diagnosis is vital, especially for conservative treatment cases.

In previous studies [1–5], mismatch repair (MMR) protein expression was preserved in most CHEC tumors. A few cases had aberrant staining of p53. The glandular epithelium usually demonstrated moderate to strong staining of ER and PR, while the squamous and corded components displayed negative or weak expression of ER and PR. Nuclear staining of beta-catenin expression can be shown in the glandular, squamous, and corded components of the majority of cases. In molecular analysis, pathogenic CTNNB1 mutations involving exon 3 were identified in all 14 patients with CHEC [4, 5], and the majority of the 14 cases were considered as copy number low, except for two P53 mutation cases, according to the Cancer Genome Atlas (TCGA) molecular classification of endometrial cancer. Our study did not includemolecular analysis due to limitations at that time in our hospital. MMR protein expression was positive in our case. ER and PR staining was strongly positive in the epithelium and negative in the sex-cord area and P53 expression was wild type. Immunohistochemistry indexes were similar to those reported in previous cases.

Molecular classification is an important factor for prognostic assessment and selection of adjuvant treatment options in patients undergoing radical surgery for endometrial cancer [12]. However, the clinical value of different molecular typing for fertility preservation therapy in patients with EC/EAH remains unclear. Reports on whether MMR-d or NSMP molecular typing affects patients’ with EC/EAH conservation outcomes are inconsistent. Our data [13] showed that the CR rate of patients with MMR-d and NSMP was close to 90%, and there was no statistical significance. However, patients with MMR-d had a higher risk of recurrence than patients with NSMP after CR [14]. Patients with p53mut type have a high risk of invasion and are thought to not be suitable for fertility preservation therapy. In addition, a small number of patients with POLE mutant may not be sensitive to progesterone, which is not consistent with other research. Molecular typing helps to screen and assess whether these patients are suitable for fertility preservation therapy. Moreover, different gene expressions may play a role too. We suspect that in the future, integrated assessment, including liquid biopsy like tumor cells DNA and serum miRNA, and gene markers, together with molecular typing and classical pathological diagnosis may be used to help make decisions in fertility-sparing management [15, 16]. More research is warranted.

Due to limited cases, data on the long-term outcomes of CHEC is rare. As indicated in the research [1, 2], the prognosis of early-stage CHEC is similar to that of typical endometrioid carcinoma. Similarly, in another study [4], stage IA CHEC cases harbored favorable clinical outcomes, while IB to IIIA CHEC cases showed aggressive clinical courses and unfavorable outcomes. All of the above data is from patients with staging surgery. The prognosis of CHEC cases taking conservative treatment requires additional research.

Tracking newborn outcomes following conservative treatment of endometrial cancer is also necessary. Our patient became pregnant naturally, and no signs of fetal chromosomal abnormalities or structural abnormalities were found during pregnancy and post-delivery. The growth curve of the fetus was within the normal range. The Extended Noninvasive Prenatal Screening (NIPT-Plus) test was completed at 17 weeks of pregnancy, indicating a low risk.

We retrospectively reviewed this case and speculated that several factors may have attributed to the success. First, the sex cord structure was local and the lesion was local without any evidence of myometrium invasion. Second, the patient had no metabolic abnormalities or other infertility factors. Third, the patient was reliable and took regular inspections. We did not expect successful natural pregnancy given that the AMH value was considerably low. Furthermore, we applied typical conservative treatment of low-grade, presumed stage IA EEC for this CHEC case because no standards were available for reference. From this case, we believe that highly selected CHEC cases should not be rejected from conservative treatment; however, caution is warranted and further exploration is needed, including exploration of the best clinical practices, regimens, monitoring protocols, and maintenance treatment after CR.

## Limitations

This study presents a case report. The follow-up time was not long, which may have limited our assessment of recurrence and long-term prognosis. We will continue to follow up. Additionally, molecular classification was not performed in this case due to technical limitations at that time.

## Conclusion

In conclusion, this is the first attempted case of fertility-sparing treatment for CHEC, and fortunately, it led to a spontaneous pregnancy and normal delivery. For highly selected, young, nulliparous patients with CHEC who have a strong desire for fertility, conservative treatment may be an option.

## Data Availability

The datasets generated for this study are available upon request to the corresponding author.
